# Living Fungi in an Opencast Limestone Mine: Who Are They and What Can They Do?

**DOI:** 10.3390/jof8100987

**Published:** 2022-09-20

**Authors:** Chakriya Sansupa, Witoon Purahong, Ali Nawaz, Tesfaye Wubet, Nakarin Suwannarach, Panuwan Chantawannakul, Sutthathorn Chairuangsri, Terd Disayathanoowat

**Affiliations:** 1 Department of Biology, Faculty of Science, Chiang Mai University, Chiang Mai 50200, Thailand; 2 Department of Soil Ecology, UFZ-Helmholtz Centre for Environmental Research, 06120 Halle (Saale), Germany; 3 Department of Community Ecology, UFZ-Helmholtz Centre for Environmental Research, 06120 Halle (Saale), Germany; 4 Department of Civil, Geo and Environmental Engineering, Technical University of Munich, Am Coulombwall 3, 85748 Garching, Germany; 5 German Centre for Integrative Biodiversity Research (iDiv), Halle-Jena-Leipzig, 04103 Leipzig, Germany; 6 Research Center of Microbial Diversity and Sustainable Utilization, Chiang Mai University, Chiang Mai 50200, Thailand; 7 Research Center in Bioresources for Agriculture, Industry and Medicine, Chiang Mai University, Chiang Mai 50200, Thailand

**Keywords:** fungi, fungal community, limestone quarry, mine rehabilitation, restoration, microbial function, soil functions

## Abstract

Opencast limestone mines or limestone quarries are considered challenging ecosystems for soil fungi as they are highly degraded land with specific conditions, including high temperature, prolonged sunlight exposure, and a lack of organic matter, moisture, and nutrients in soil. In such ecosystems, certain fungi can survive and have a crucial function in maintaining soil ecosystem functions. Unfortunately, we know very little about taxonomic diversity, potential functions, and the ecology of such fungi, especially for a limestone quarry in a tropical region. Here, we characterized and compared the living soil fungal communities in an opencast limestone mine, including mining site and its associated rehabilitation site (9 months post-rehabilitation), with the soil fungal community in a reference forest, using the amplicon sequencing of enrichment culture. Our results showed that living fungal richness in the quarry areas was significantly lower than that in the reference forest, and their community compositions were also significantly different. Living fungi in the mining sites mostly comprised of Ascomycota (Eurotiomycetes and Sordariomycetes) with strongly declined abundance or absence of Basidiomycota and Mucoromycota. After nine months of rehabilitation, certain taxa were introduced, such as *Hypoxylon* spp. and *Phellinus noxius*, though this change did not significantly differentiate fungal community composition between the mining and rehabilitation plots. The majority of fungi in these plots are classified as saprotrophs, which potentially produce all fifteen soil enzymes used as soil health indicators. Network analysis, which was analyzed to show insight into complex structures of living fungal community in the limestone quarry, showed a clear modular structure that was significantly impacted by different soil properties. Furthermore, this study suggests potential taxa that could be useful for future rehabilitation.

## 1. Introduction

Mining is considered an excessive ecosystem disturbance. Complete removal of vegetation and soil layers during mining activities remarkably changes the ecosystem in terms of both environmental condition and resources, causing loss in biodiversity and important soil elements [1,2,3]. According to Bell et al. [4], a mining site is considered the severest form of land degradation, requiring an intensive restoration practice to restore biotic and abiotic factors. One of the critical points to consider concerns the ability of the living organism to survive in the harsh condition of the mining area.

An opencast limestone mine or limestone quarry is a challenging habitat for all living organisms. The operation of a limestone quarry increases drainage and the physical and chemical erosion of the substrate, hindering natural germination and plant re-establishment [5]. A lack of soil organic materials, soil moisture, macro- (N, P, K), and micro-nutrients (Fe, Mn), and soil compaction were the general issues in the limestone quarry [6]. In this study, we referred these issues as “harsh/extreme condition.” The harsh condition of a limestone quarry allows only few plants and animals species to naturally re-establish in the site [5,6,7]. In addition, several soil microbes may largely disappear. For soil bacteria, it has been demonstrated that community composition and diversity were significantly altered in the mining site when compared to the reference pre-mined site (which is usually a forestland) [6,8]. Specifically, a study on living bacteria in limestone quarry presented potential bacterial taxa inhabiting the quarry area, proposing that spore producing bacteria could be promising taxa, tolerating the extreme mine conditions and can be of particular interest for further mine rehabilitation [6]. However, such data is still unclear for fungi. Therefore, this presented study was carried on the same study site to observe the information on fungi. Particularly, living fungal community, which potentially performed soil functions, are of interest as they are resistant to the difficult conditions of the quarry and may help in the early phase of rehabilitation processes.

Fungi are among the most significant microorganisms in soil systems [9]. According to Frąc, et al. [10], fungi operate as three major ecosystem drivers to sustain soil health: decomposers, ecosystem regulators, and biological controllers. Furthermore, due to the ability of fungi to take on numerous forms, they can successfully inhabit a variety of soil conditions, even the unfavorable [10]. They can also function as ecological regulators, responsible for soil formation, rock weathering, or organic compound decomposition [10,11]. Certain fungi may act as biological controllers, assisting in disease control or promoting the growth of other species, especially plants [12,13,14]. When it comes to mine restoration, observation of soil fungi that can live in the harsh condition of mining areas is important, since a previous study has shown that exotic microorganisms fail to grow in mine substrate and could not promote rehabilitation outcomes [15]. Thus, observing fungal variety under harsh conditions of an opencast limestone mine/limestone quarry may aid in the development of a better rehabilitation plan. Specifically, living fungi, which includes active, potentially active, and dormant cell [16], that are easily cultured are of interest as they can be used for future work.

This study was conducted on an opencast limestone mine in Northern Thailand. Soil samples were collected from three sites, including forest, mining, and young mine rehabilitation plots. We aimed to (i) compare living fungal community in a mining site, young mine rehabilitation plot, and adjacent remnant forest, (ii) identify the living and culturable fungi that survived in an opencast limestone mine and their potential functions for mine rehabilitation, (iii) investigate the environmental factors shaping the living fungal community composition and (iv) explore co-occurrence networks between living fungi found in the harsh environment of mining and young mine rehabilitation plots. This work combined enrichment culture with culture-independent molecular approaches modified from Sansupa et al. [17] to identify the living fungal diversity and their associated functions. The method can provide data on culturable fungi that could be used as material for future rehabilitation practice.

## 2. Materials and Methods

### 2.1. Site Description and Soil Physicochemical Properties

This study was carried out at a semi-opencast limestone mine/limestone quarry in Lampang Province, Northern Thailand (18°32′23″ N, 99°34′47″ E). Air temperature and rainfall data were collected from a weather station of the Thai Meteorological Department—Lampang. The average annual temperature was 27.2 °C and mean annual rainfall was 3.6 mm. (2016–2018).

**Figure 1:**
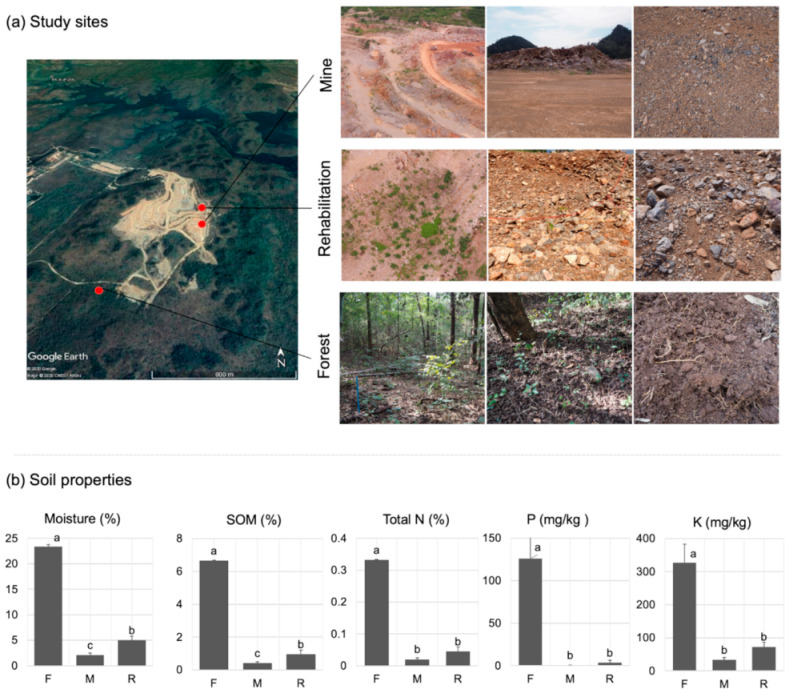
Study sites and soil physicochemical properties. (**a**) Study site locations with an overview of the semi-opencast limestone mine and photos of three study sites including natural forest (F), mining site (M), and rehabilitation plots (R); (**b**) Soil physicochemical properties, including moisture, soil organic matter (SOM), total nitrogen (Total N), phosphorous (P), and potassium (K). Different lowercase letters (a, b and c) indicate significant differences.

Soil samples were collected from three study sites in and around the quarry (Figure 1a). These included (i) Forest (F): natural bamboo-deciduous forest located adjacent to the mining site. This forest is covered by a mix of deciduous tree species and a dense thicket of bamboo (*Bambusoideae*) [18]. Tree species that were commonly found in this area included but are not limited to *Antidesma sootepense*, *Phyllanthus emblica*, *Pterocarpus macrocarpus* and *Tamilnaldia uliginosa*. A list of tree species found in the forest were shown in Table S1. This forest was used as a reference/goal for a rehabilitation project since the plant community and environmental conditions are more or less similar to the pre-mined sites. (ii) Mining site (M): site located around the main pit perimeter. The mine was dug deep to sub-soil layer to collect commercial minerals. Plants and topsoil were all removed from this area, leaving only a sub-soil layer on top of the limestone quarry floor. The floor was hard packed with rock and gravel. (iii) rehabilitation plots (R): the small plots located around the main pit. The rehabilitation procedure has been processed for 9 months. The procedure included the dumping of sub-soil stockpiles and subsequent planting of the 30–50 cm tall saplings. Sub-soil stockpile was loosened substrate that had been left over from operation of the quarry; it lacked organic matter that could be characterized as topsoil but was loose enough to enable water, oxygen, and plant roots to penetrate the ground. Planted tree species were selected according to the framework tree species method, which is a selection of tree species (from referenced forest) able to accelerate forest regeneration [19]. The tree species that were planted in the rehabilitation site included but were not limited to *Gmelina arborea*, *Antidesma sootepense*, *Pterocarpus macrocarpus*, and *Ficus racemosa*. All planted tree species can be found in Table S2.

Soil physicochemical properties, including soil organic matter, soil texture, macro-and micro-nutrients of the study area, were measured and presented in Sansupa et al. [6]. Briefly, forest soil was considered as clay (10% sand, 13% silt, 77% clay) with pH (H_2_O) 7.79 and contained a higher level of organic matter (6.65% SOM) and nutrients (i.e., 0.33% total N, 126 mg/kg P and 326 mg/kg K) than mine and rehabilitation plots (Figure 1b). On the other hand, soil texture in mine and rehabilitation area is considered as sandy loam (64% sand, 18% silt, 18% clay) with pH (H_2_O) 8.82 and sand clay (47% sand, 18% silt, 35% clay) with pH (H_2_O) 8.55, respectively. The mine substrate contained 0.42% SOM, 0.02% total N, < 0.05 mg/kg P and 33.20 mg/kg K, whereas rehabilitation substate contained 0.94% SOM, 0.05% total N, 3.44 mg/kg P and 72.57 mg/kg K (Figure 1b). In the forest, mine, and rehabilitation plots, soil moisture was approximately 23%, 2%, and 4%, respectively (Figure 1b). Methods used to measure these soil properties are presented in Table S3.

### 2.2. Sample Collection

In each study site, 5 square plots (5 m × 5 m) were set up at a minimum distance of 20 m apart. Each square plot represents a biological replication in the study sites (*n* = 5). Soil samples were taken in June 2018. A total of 5 subsamples were collected to 10 cm depth, using an auger 10 cm in diameter. The subsamples were bulked into one composite sample and filtered through a sieve of 2 mm. These samples were kept in an icebox during transportation and subsequently used for the living fungal identification method within 24 h.

### 2.3. Identification of Living Fungal Community by Amplicon Sequencing of Enrichment Culture

The samples collected from limestone quarry in this study did not provide a high quality and quantity of eDNA and the amplification of microbial gene failed [6]; thus, total fungal diversity cannot be investigated by amplicon sequencing of the eDNA. With the limitations, we enriched the soil fungal community on culture media before taxonomical identification by amplicon sequencing. The amplicon sequencing of enrichment culture method, as previously described by Sansupa et al. [17], was employed in this study. Although, it is noted that this method provided only the viable and culturable part of the community, it was proposed as an alternative method for low microbial abundance samples and can reveal both dominant and rare taxa in the total community [6,17,20]. However, as the original method was demonstrated on bacteria, necessary modifications that included changing culture media and increasing incubation time, were made to encourage fungal growth. In detail, a living fungal community was identified in the following steps.

#### 2.3.1. Enrichment of Soil Fungi

Soil fungi were enriched by three culture media including, potato dextrose agar (PDA), yeast malt agar (YM), and Dichloran Rose Bengal Chloramphenicol (DRBC) agar. In detail, 1 g of soil was added to 9 mL sterilized 0.85% NaCl and shaken thoroughly. A 100 μL of soil suspension was added to culture media and incubated at 25 °C for 7 days. Subsequently, all colonies grown on each culture media were collected and mixed in one collection tube. The colony mixtures were kept at −20 °C until further analyses.

#### 2.3.2. DNA Extraction and Amplicon Sequencing

DNA was extracted from 300 μL of the colony mixture using a NucleoSpin^®^ Soil DNA extraction kit, according to the manufacturer’s instructions. DNA samples were then amplified at internal transcribed spacer 2 (ITS2) region using forward primer ITS3F 3′-GCATCGATGAAGAACGCAGC-5′ and reverse primer ITS4R 3′-TCCTCCGCTTATTGATATGC-5′ [21]. Sequencing was performed using an Illumina MiSeq platform. The amplification and sequencing steps were performed at Macrogen, South Korea. Raw sequence datasets are available in the National Center for Biotechnology Information (NCBI) under BioProject accession number PRJNA753229.

#### 2.3.3. Sequencing Data Analysis

Sequencing data analysis was performed using MOTHUR [22] and the Standard Operating Procedure (SOP) custom-analysis work-flow [23]. Raw sequence reads with a minimum overlap at 20 nucleotides were assembled using simple Bayesian algorithm (threshold = 0.6) as implemented in PANDAseq [24]. The assembled reads were then filtered for high-quality reads (length ≥ 200 nucleotide, Phered score ≥ 20). The chimeric sequences were detected using the UCHIME algorithm [25] and removed from the dataset. The non-chimeric datasets were clustered at 97% similarity into operational taxonomic units (OTUs) using CD-HIT-EST algorithm [26] and assigned taxonomy based on UNITE database v.7 [27] using the naive Bayesian classifier [28]. After that, rare OTUs, including singletons, doubletons, and tripletons, were removed. The datasets were normalized to 65,000 reads/sample using “rrarefy” in vegan package [29] and used for further analyses.

#### 2.3.4. Functional Prediction

The potential functions associated with fungi were predicted using FungalTraits [30] and PICRUSt2 [31]. The FungalTrait database contains 10,210 fungal genera, which are classified into 17 cryptic lifestyles/traits. The fungal lifestyles/traits were assigned based on genus level. On the other hand, the PICRUSt2 predict functional potential of fungi based on marker gene sequencing profile. The gene families for ITS sequencing data were annotated corresponded to Enzyme Classification numbers (EC numbers). Specifically, this study emphasized the potential enzyme activity (based on detected gene families) of 15 soil enzymes, which could be an indicator for soil health [32], including Acid phosphatase, Alkaline phosphatase, Alpha-amylase, Alpha-N-acetylglucosaminidase, Amidase, Arylsulfatase, Beta-glucosidase, Cellulase, Chitinase, Endo-1,4-beta-xylanase, Laccase, Pectin lyase, Peroxidase, Urease and Xylan 1,4-beta-xylosidase.

### 2.4. Statistical Analysis and Co-Occurrence Network Analysis

All statistical analyses were performed in PAST [33] and R programming [34]. The significant differences of fungal communities in forest, mine, and rehabilitation plots were tested using nonparametric multivariate analysis of variance (NPMANOVA). Fungal community composition based on abundance (Bray–Curtis dissimilarity), and presence/absence (Jaccard dissimilarity measure) data were visulised by Non-metric multidimensional scaling (NMDS) ordinations. Subsequently, soil physicochemical parameters were fitted to the fungal community composition using “envfit” in vegan package. Goodness-of-fit (*R*^2^) and significant value (*p*-value) were presented in Table S4. The significant parameters (*p* < 0.05) were included in the ordination plots. Furthermore, the functional profiles based on all predicted enzyme activity were visualized by principal component analysis (PCA). Differences in the functional profiles among the three study sites were tested by NPMANOVA. Moreover, the difference between 15 important enzyme activities in the 3 study sites were tested using ANOVA (where data were normally distributed) or Kruskal–Wallis (where data were not normally distributed). Besides, the difference between numbers of OTUs associated with each fungal trait (OTUs rich Traits) in each study site was tested by ANOVA.

Co-occurrence network analysis was performed to observe the relationships of fungal community in extreme environment (mine and rehabilitation plots). Spearman-rank correlations were calculated between all fungal OTUs detected in the extreme environment. Correlation coeffeicient (σ) more than 0.7 with a significant *p*-value (*p* < 0.05) were considered as robust and used to generate co-occurrence network. The network properties was caculated with igraph package [35] and subsequently visualized by Gephi [36] using an undirected network and Frauchterman–Reingold layout. In this network analysis, node represents OTUs, while edge/link represents the connection between OTUs. Nodes were grouped into modules when they were highly connected within their own group but much less connected outside the group [37]. A network has a modular structure when the modularity value of the network is greater than 0.4 [37]. In this study, modules of less than five nodes were excluded.

To identify node topologies, node connectivity within a module (Z_i_) and between module (P_i_) were calculated using “gateway_coeff” and “part_coeff” function in brainGraph package [38]. The node topologies were classified based on Z_i_ and P_i_ coefficient, into four simple catagories including, peripheral nodes (Z_i_ ≤ 2.5, P_i_ ≤ 0.62; nodes with a few edges), connectors (Z_i_ ≤ 2.5, P_i_ > 0.62; node links to several modules), module hubs (Z_i_ > 2.5, P_i_ ≤ 0.62; node links to many nodes in their own modules), and network hubs (Z_i_ > 2.5, P_i_ > 0.62; node links to many nodes both inside and outside their own modles) [39,40]. The network properties, Z_i_ and P_i_ score were presented in Table S5. Furthermore, the fungal community composition of all living fungi in extreme environments and that of each module were analyzed with PCA for those composition with short gradient length or CA for those composition with long gradient length. Correlation between three axis score (axis 1, axis 2, and axis 3), deriving from PCA or CA, of each community and soil physicochemical properties were calculated using Spearman-rank correlation.

## 3. Results

### 3.1. General Infomation: An Overview of Sequencing Analysis

A total of 1,550,544 high-quality reads were detected in this study. Specifically, 636,195 (127,239 ± 9903, mean ± SE), 478,192 (95,638 ± 8131) and 436,157 (109,039 ± 16,034) reads were detected from forest, mining, and rehabilitation plots, respectively. After normalization and taxonomic classification, 350 fungal OTUs were obtained. Futhermore, rarefaction curves of the fungal OTUs derived from the three study sites gradually reached the saturation stage indicating that the detected OTUs were sufficient to represent the living fungal richness and community composition in this study (Figure S1).

### 3.2. Living Fungal Community Composition: The Distribution of Living Fungi in Mining and Rehabilitaion Plots as Compared to Forest

**Figure 2:**
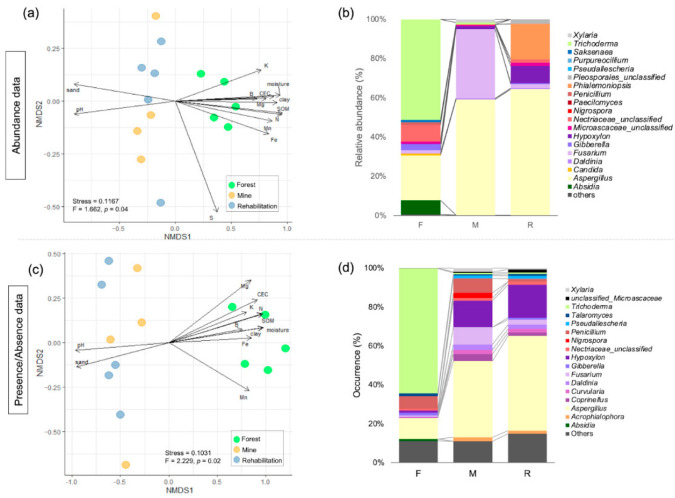
Fungal diversity and taxonomic distribution across three study sites, including forest (F), mining (M), and rehabilitation plots (R): fungal community compositions based on (**a**) Bray–Curtis and (**c**) Jaccard dissimilarity measure and taxonomic distributions based on (**b**) abundance and (**d**) presence/absence data.

Fungal richness in mining and rehabilitation plots were significantly lower than that in the forest. Whilst 82 OTUs (26 ± 4, mean ± SE) and 88 OTUs (31 ± 2, mean ± SE) were respectively found in mining and rehabilitation plots, 265 fungal OTUs (105 ± 5, mean ± SE) were found in the forest. There were three fungal phyla, including Ascomycota, Basidiomycota, and Mucoromycota, detected in this study (across three study sites), but the latter was only detected in the forest. Whilst the most abundant and frequently occurring taxa in the forest were Sordariomycetes, Eurotiomycetes, Mucoromycetes, and Dothideomycetes, those in the mining and rehabilitation plots were Eurotiomycetes and Sordariomycetes. Significant disimilarlities were found in the fungal community compositions in the three study sites. According to abundance data (based on Bray–Curtis dissimilarity measure), the composition of the living fungal community in mining and rehabilitation plots were similar. Abundance taxa in the mine were *Aspergillus* (59%), *Fusarium* (35%), *Hypoxylon* (1.6%), and *Xylaria* (1.6%), whereas those in rehabilitation plots were *Aspergillus* (64%), *Phialemoniopsis* (18%), *Hypoxylon* (8%), and *Fusarium* (2%) (Figure 2). On the other hand, the community compositions in mining and rehabilitation plots were both significantly different from that in the forest (*F* = 1.662, *p* = 0.04; Figure 2a). Fungi belonging to *Trichoderma* (51%), *Aspergillus* (23%), *Nectriaceae* (8%), and *Absidia* (7%) dominated in the forest (Figure 2b).

Nevertheless, fungal community compositions based on presence/absence data were slightly different from those based on abundance data. The presence/absence data illustrated that the community composition of living fungi in the forest is significantly different from those in mining and rehabilitation plots, but again no differences were found between mining and rehabilitation plots (*F* = 2.229, *p* = 0.02; Figure 2c). Whilst the most frequently occurring taxa in mining site were *Aspergillus* (39%), *Fusarium* (9%), *Penicillium* (7%), and *Coprinellus* (3%), those in rehabilitation plots were *Aspergillus* (49%), followed by *Hypoxylon* (17%), and *Fusarium* (2.6%) (Figure 2d). On the other hand, the frequently occurring taxa detected in the forest belonged to *Trichoderma* (65%), *Aspergillus* (11%), and *Penicillium* (6%) (Figure 2d).

In addition, we found that several taxa (45 OTUs; i.e., *Curvularia affinis*, *Fusarium solani*, *Aspergillus niger*, *Trichoderma* sp., and *Hypoxylon* sp.) were found in all study sites, while many taxa that were detected in the forest disappeared in mining and rehabilitation plots, for example, *Trichoderma* spp., *Candida glabrata*, and *Saksenaea oblongispora*. Moreover, several taxa were only detected in the limstone quarry area (Mining and Rehabilitation), for example, *Aspergillus amstelodami*, *Aspergillus fumigatus*, *Humicola phialophoroides*, *Curvularia clavata*, and *Phellinus noxius* (Table S6). When it comes to fungal taxa in limestone quarry sites, it is also noticed that certain taxa found unique in mine sites, for example *Aspergillus alabamensis* and *Nigrospora oryzae*, and rehabilitation plots, for example *Gibberella intricans*, *Hypoxylon* spp., and *Phellinus noxius* (Table S6). However, this difference was small and did not significantly differentiate overall community composition between the two sites. The results of soil physico-chemical properties fitted to the ordinations illustrated that several parameters significantly influenced the living fungal community compositions (Figure 2a,c; Table S4). Whilst percentage of sand and pH were positively correlated to the fungal communities in mine and rehabilitaion plots, moisture, SOM, and nutrients (i.e., N, P, and Mn) were positively correlated to the community in the forest (Figure 2a,c).

### 3.3. Predictive Functional Profiles: Fungal Traits and Enzyme Activities Detected in Mining and Rehabilitaion Plots as Compared to Forest

Two functional prediction tools were used in this study. In total, 334 out of 350 fungal OTUs were identified into a total of 11 fungal traits, including saprotrophs (unspecified saprotroph, soil saprotroph, litter saprotroph, wood saprotroph, nectar/tap saprotroph, dung saprotroph), animal parasite, epiphyte, foliar endophyte, mycoparasite, and plant pathogen. However, there were only three main traits, including mycoparasite, saprotrophs, and plant pathogen, that contained more than five associated OTUs. On the other hand, 254 out of 350 fungal OTUs were assigned to 897 enzymes, and 15 selected soil enzymes were found in all study areas.

**Figure 3:**
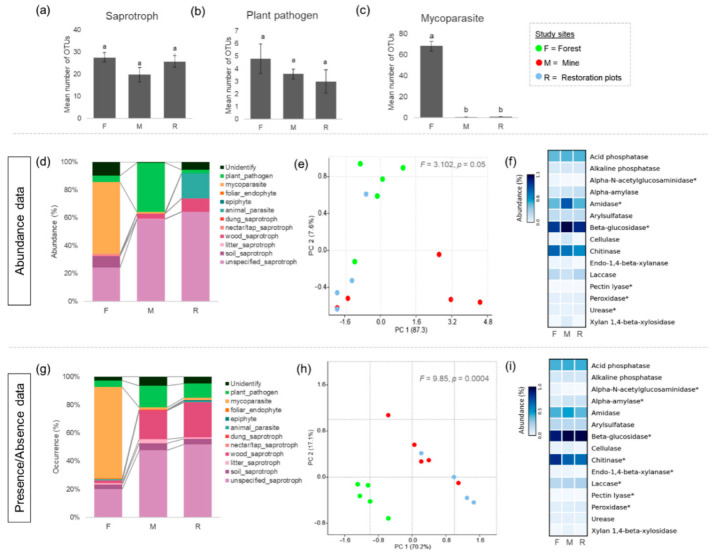
Functional profile predicted by FungalTraits and PICRUSt2. Bar plot shows the average number of OTUs ascribed to different fungal traits, including (**a**) Saprotroph (**b**) Plant pathogen and (**c**) Mycoparasites. Stack bar plots present fungal traits distribution among three study sites based on (**d**) abundance data and (**g**) presence/absence data. PCA ordinations show the composition of PICRUSt2-predicted enzymes based on (**e**) abundance OTU data and (**h**) presence/absence OTU data. The average number of 15 important soil enzymes based on (**f**) abundance OTU data and (**i**) presence/absence OTU data were shown in heatmap. Enzymes with asterisk present statistically significant (*p* < 0.05). Different lowercase letters (a and b) indicate significant differences.

In the harsh conditions of the quarry sites (mining and rehabilitation plots), more than 90% of fungal OTUs were assigned to at least one trait. Whilst the most abundance traits were saprotrophs and plant pathogens, followed by animal parasite, frequently occurring traits were saprotrophs and plant pathogens, followed by mycoparasite, epiphyte and animal parasites. However, only 2 traits that contain more than 5 OTUs were saprotrophs (93 OTUs) and plant pathogens (16 OTUs). According to statistical analysis, the OTUs rich Trait (number of OTUs associated with particular function/trait) of saprotrophs and plant pathogen in mining and rehabilitation plots were not significantly different from that in the forest (Figure 3a,b). However, OTUs rich Trait of mycoparasite in the forest is significantly greater than mining and rehabilitation plots (Figure 3c). The most prevalant traits in the forest were mycoparasite, followed by saprotrophs and plant pathogens (Figure 3d,g).

A total of 897 enzymatic genes (that associated with fungal sequences), predicted by PICRUSt2, were detected. The PCA ordination considering functional profile based on the abundace of all detected enzymes presented no significant difference among the three study areas (Figure 3e). In contrast, a difference was found between the predicted functional profiles based on presence/absence OTUs data. The functional profiles in the mining and rehabilitation plots were dissimilar from those in the forest (Figure 3h). Furthermore, 15 enzymes reported as the important indicators of soil health were selected to illustrate soil functional performance of the fungi in each study site. The results showed that all 15 enzymes were found to be possibly secreted by living fungi detected in all study sites. Specifically, we found that certain enzymes, such as Beta-glucosidase, tend to have higher activity in the harsh environments of the limestone quarry as compared to the forest. However, some enzymes, such as Alpha-N-acetylglucosaminidase and Chitinase, may have more activity in the forest than in mining and rehabilitation areas (Figure 3f,i).

### 3.4. Fungi in Harsh Condition of Limestone Quarry: Taxonomic Distribution and Co-Occorence Network of Living Fungi in the Mining and Rehabilitatoin Plots

#### 3.4.1. Living Fungi in Mining and Rehabilitation Plots

In this study, mining and rehabilitation plots were considered as areas with harsh soil conditions as there were conditions of drought, heat exposure, and poor soil nutrition. Here, 130 fungal OTUs, belonging to Ascomycota and Basidiomycota were discovered. The most abundant taxa found in both mining and rehabilitation plots were Sordariomycetes and Eurotiomycetes. On the other hand, the most frequently occurring taxa (presence/absence data) belonged to Eurotiomycetes, Sordariomycetes, Dothideomycetes, and Agaricomycetes. *Aspergillus* species dominated. Fungal taxa found in these area were listed in Table 1 (and Table S6). Here, to highlight the potential use of fungi for mine rehabilitation purpose, we listed fungal taxa found in the limestone quarry and added details on the plant-related functions based on prior studies. Furthermore, the potential strategies to survive in the harsh conditions of the limestone quarry are also presented. However, notice should be taken that the stress response information of several detected taxa in this study was not shown because there were no previous reports on such topic (Table 1).

#### 3.4.2. Co-Occurrence Network and Taxonomic Distribution of Living Fungi in Mining and Rehabilitation Plots

**Figure 4:**
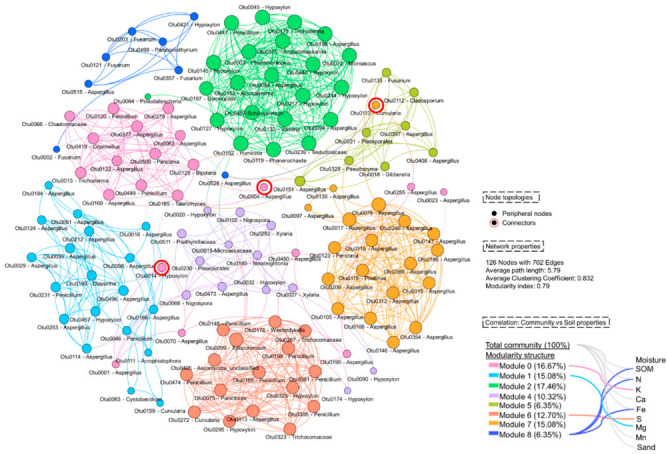
Co-occurrence network of living fungi found in limestone quarry sites (mining and rehabilitation plots). A links/edge stands for a significant correlation based on Spearman’s rank correlation (σ > 0.7, *p* < 0.05). Node size corresponding to degree. Node color corresponding to modularity class. The links between modular community and soil properties represent the significant correlation based on Spearman’s rank correlation (σ > 0.7, *p* < 0.05).

To determine the insightful structure of fungi in the harsh environment of the limestone quarry, co-occurrence network, network properties, and critical hubs were calculated and identified. In detail, the network consisted of 126 OTUs (nodes) with 702 connections (edges) (Table S5). The average path length and average clustering coefficients were 5.79 and 0.832, respectively. A modularity index of the network was 0.79, which suggested that the network had a modular structure. Specifically, nine modular communities were generated in this study (Figure 4). Whilst Module 0, Module 1, and Module 7 were dominated by *Aspergillus* species, Module 2, Module 6, and Module 8 were dominated by *Hypoxylon*, *Penicillium*, and *Fusarium*, respectively. Based on Z_i_ and P_i_ scores, there were three nodes, belonging to OTU0004 (*Aspergillus flavus*), OTU0014 (*Hypoxylon* sp.), and OTU0113 (*Curvularia affinis*) that were found to be connectors of this network. Other nodes were classified as peripheral nodes. Furthermore, we found that each modular community was significantly correlated with different soil properties (σ > 0.7, *p* < 0.05; Figure 4). Total fungal community in this area was significantly correlated to SOM, N, Ca, Fe, Mn, and percentage of sand (Figure 4).

Taxonomic distribution based on the 126 nodes on the co-occurrence network illustrated that 2 phyla, including Ascomycota and Basidiomycota, were found. Fungal genera that frequently occurred were *Aspergillus* (38.68%), *Hypoxylon* (11.90%), *Penicillium* (9.53%), *Fusarium* (3.97%), and *Curvularia* (2.38%) (Figure S2). The most frequently detected functional trait belonged to saprotroph (75.39%), followed by plant pathogen (12.7%) and mycoparasite (3.17%; Figure S2).

## 4. Discussion

The operation of an opencast limestone mine or limestone quarry had a significant impact on edaphic parameters and microbial communities. Taxonomic distribution and community composition of soil microbe, including both bacteria and fungi, were intensively disturbed by the processes [6,8]. This study illustrated living fungi response to the mining operations and early rehabilitation process. This is a case study, which carried on an opencast limestone mine in Northern Thailand and it is noted that this study can only present the living and culturable fungal community. Here, we revealed that mining decreases living fungal diversity, compared to the reference forest. No significant difference were found on overall living fungal community composition between mining area and rehabilitation plots. However, this study highlights the taxonomic distribution of living fungi detected in the limestone quarry and discusses their living strategies, potential function, and benefit to plants, which could be promising information for future rehabilitation plans.

### 4.1. Living Fungi and Early Mine Rehabilitation: Community Structure, Diverity and Their Associated Function on Early Rehabilitaion Plot as Compared to Mining and Forest Sites

This study revealed that there is no respose on the live fungal community in the early stages of mine reahbilitation (<1 year). The living fungal community in rehabilitation plots was comparable to that of the mining site, but considerably different from that of forest soil. This is consistent with the trend seen in living bacterial community of the same site [6]. In this case, the living bacterial and fungal communities did not provide an immediate response to mine rehabilitation. However, it should be noted that the technique utilized was limited in that only culturable fungi were discovered. Such results are unclear when it come to the eDNA-based method. To date, there has been relatively little comparison of fungal communities in young mine restoration plots and un-restored mine sites. More research is needed to answer the question of how long the restoration time should be to dramatically alter the soil microbial community in the restoration plot relative to the un-restored mine area. These could show the effect of restoration in an early age. On the other hand, several studies have been conducted on fungal community response to restoration chronosequence. Whlist Ngugi et al. [62] demonstrated that fungal communities eventually became more similar to the reference condition over time, Kane et al. [63] found no obvious trend in fungal diversity toward the reference forest. However, both investigations found that the fungal community was mostly shaped by edapic variables [62,63]. Consistently, our research revealed that soil organic matter, pH, texture, moisture, and nutrients influenced live fungal communities, with the communities in mining regions different from those in the forest. However, a little increase in soil organic matter in the restoration plot was not enough to distinguish its fungal community from that of the un-restored mine site. Still, there are some taxa, for example, *Hypoxylon* spp. and *Phellinus noxius*, that were discovered in the restoration plot but not at the mining site. These might be caused by the introduction of sub-soil stockpile and planted trees.

Based on funtional prediction tools used in this study, saprophytic fungi were abundant in all study sites, but they were especially abundant in mining and rehabilitation plots (almost 50% of all detected traits). The result was consistent with a previous study on sand mine restoration [64]. According to Naranjo-Ortiz and Gabaldón [65], saprophytic fungi can thrive in a variety of niches and are frequently found in harsh environments. This may be explained by the abundace of saprophytic fungi in the quarry sites: since quarries are specific habitats with no plant cover, low soil nutrients and low organic substrate, other fungal lifestyles, except for saprotrophs, may not be able to survive in such conditions. In contrast, a high abundance of saprophytic fungi and mycoparasite and some epiphyte, endophyte, and plant pathogens were detected in the forest. Since the forest contained multiple habitats for various fungal species, it is not surprising that several fungal traits were detected. However, it is noticed that mycorrhizae, which are important fungi in the soil ecosystem, were not found in this study and this may be caused by the limitation of the culture method employed in this study. More diverse culture media and longer incubation periods may increase the number of fungal taxa detected, increasing the method’s effectiveness [17]. Furthermore, it should be noted that functional assignment in this study may not cover all possible functions contributed by the community. Previous studies have demonstrated that fungal functions, predicted by PICRUSt2 and FungalTrait, may be limited by the number of genes available in the database and poor taxonomic classification [30,31,66].

### 4.2. Living Fungi in Harsh Condition of the Limestone Quarry: There Are Many Way to Survive

The extreme condition in this study relates to drought, heat exposure, low nutrient availability, and slightly alkaline (~ pH 8) soil, which were all caused by mining operation. We demonstrated that Ascomycota fungi, mainly Dothideomycetes and Eurotiomycetes, are often found in this area. This could be attributed to the fact that Ascomycota is a widespread and ubiquitous phylum that represents the most diffuse group of fungi and has the greatest number of currently recognized species [67]. However, our finding follows a similar pattern to earlier research that found fungi belonging to these two classes predominating in dry [68] and alkaline [69] environments. Despite the fact that this study cannot determine whether the identified taxa were active or dormant in such conditions, it is believed that detected taxa were able to survive under drought and low nutrient soil due to the utilized method. We demonstrated that fungi belonging to the *Aspergillus* species were the most abundant taxa found in the severe conditions of the opencast limestone mine. This corresponds with previous study that found *Aspergillus* DNA in a variety of severe environments, such as high salinity and cold [43]. The presence of *Aspergillus* species in severe environments may be explained by the availability of conidia, which are asexual spores produced by Ascomycota under unfavorable conditions. It has been demonstrated that fungal conidia can withstand a variety of environmental stressors, such as drought, high temperatures, and ultraviolet (UV) irradiation [68]. These may be induced by the accumulation of compatible solutes (i.e., manitol or trehalose) in fungal conidia, which function as a cell defender under stress [69,70]. However, Wyatt et al. [68] stated that Ascospore, an Ascomycota sexual spore, is more resistant to stress than conidia. Therefore, it could imply that one of Ascomycota fungi’s strategies for survival under environmental stress is through their spores. This might be one of the reasons why 90% of the fungi detected in our study were Ascomycota. However, there may be additional strategies that can help the fungi cope with environmental stress, such as the expression of a stress response gene or the formation of thick cell walls [44,71].

### 4.3. Co-Occorence Network of Living Fungi in Harsh Condition of The Limestone Quarry: The Modular Community and Their Correlation with Soil Physiochemical Properties

According to the Zi and Pi values, no OTUs in this network were theoretically identified as hubs. However, the three connectors (OTU0004: *Aspergillus flavus*; OTU0014: *Hypoxylon* sp.; OTU0113: *Curvularia affinis)* with strong linkages to each module were discovered and might be defined as a key population of this network [72]. Furthermore, the network can be sub-structured into several modular communities. Previous studies showed that the microbial modular pattern was driven by soil physicochemical properties [73,74,75]. This was supported by our results. We demonstrated that different soil characteristics were significantly correlated with each modular community. This might suggest that members in each module have different niches [75]. Here, we showed that K, Mg, and S were all negatively correlated with Modules 0, 1, and 6, respectively. On the other hand, Module 8 was negatively correlated with SOM, Fe, and N. However, that certain modules (Module 2, Module 4, Module 5, and Module 7) did not have a significant correlation with observed soil characteristics may imply that members of these modules may exist in flexible soil conditions. However, more study is needed to clarify this issue, particularly studies that only captured active fungus.

### 4.4. Posible Interaction of Fungi and Plant: Opportunity for Rehabilitation Procedure

Most of the restoration on the quarry area has been done by covering the floor with topsoil [64,76,77]. The adding of topsoil could facilitate plant survival and act as microbial inoculant at the early stage of mine rehabilitation. This could be the priority procedure to consider when working on mine rehabilitation. However, in some cases, topsoil has not been a choice. Inoculation of plants with fungi that can both tolerate the extreme mine conditions and can promote their growth should be considered. We presented that several fungi surviving in the harsh environments of the limestone quarry were previously reported as facilitators to enhance plant-stress tolerance. For example, *Aspergillus aculeatus* and *Aspergillus fumigatus* were shown to aid plants in drought environments and to further promote their growth [42,45]. This shows a promising application of fungi detected in a mine area to accelerate plant growth and survival in a mine. However, more studies have to be done on the effects of fungal inoculation on each tree species.

### 4.5. Overall Living Fungal Community Assessment: Limitation and Recommendation for Future Work

Living fungal community in this study was investigated by amplicon sequencing of enrichment cultures. This method is beneficial for low-biomass samples where an eDNA-based method cannot be employed, as well as for rapid screening of the living microbial community and its predictive functions [17]. Although this study successfully evaluated the fungal community in each study site, it should be noted that we can only disclose culturable taxa growing in the applied media. It is recommended to apply a variety of culture media in future work to obtain more diverse taxa [17]. The identification of fungal taxa in this study based on short-read sequencing (Illumina Miseq Platform) can potentially identify the microbes deep to the genus level and, in some case, to the species level [78]. To more accurately identify the species of fungi, it is suggested to apply long-read sequencing, which was recently proposed as a promising tool for fungal species identification [79,80]. Furthermore, the application of both the traditional culture method and high-throughput sequencing would be an alternative option for future studies as it could contribute more comprehensive and better resolution in species identification and microbial distribution patterns [78,81].

## 5. Conclusions

This study reveals a living fungal community in an opencast limestone mine in Northern Thailand. The study was held in 2018, where the rehabilitation was processed for approximately 9 months. Here, we found that the rehabilitation of the opencast limestone mine, by dumping sub-soil stockpile and planting framework tree species, did not have an early (< 1 year) impact on living fungal community composition. In the limestone quarry, most of the detected fungi were saprotrophs. Ascomycota, especially *Aspergillus*, was one of the most abundant taxa that survived in the harsh conditions of this area. It is also suggesting that *Aspergillus* species could be of particular interest for future mine rehabilitation due to their ability to survive in harsh conditions and to promote plant growth. However, the plant growth information presented in this study was based on literature; further research is required to test such ability on taxa isolated from this quarry.

## Figures and Tables

**Table 1:** Living fungi detected in the opencast limestone mine.

Class	Genus	Detected Species	Stress Factors	Possible Adaptive Strategies to Extreme Environments	Application on Plant	References
Dothideomycetes	*Periconia*	unidentified	Salinity	-*	-	[41]
	Others	*Angustimassarina* *acerina*, *Curvularia* *clavata*, *Paraconiothyrium brasiliense* and *Valsaria neotropica*	-	-	-	-
Eurotiomycetes	*Aspergillus*	*Aspergillus aculeatus*	Drought	Conidia	Promote plant growth in Drought and salt stress	[42]
		*Aspergillus amstelodami*	Salinity	-	-	[43,44]
		*Aspergillus flavus*	Drought	Conidia or sclerotia	Promote plant growth under heat stress	[45,46]
		*Aspergillus fumigatus*	Drought and oxidative stress	-	Improves drought resistance	[47,48]
		*Aspergillus niger*	High temperature	Conidia	Heat-stress ameliorative tool	[49,50]
		*Aspergillus terreus*	High temperature, pH and salinity	Stress response gene	Promote plant growth and control disease	[51,52]
	*Penicillium*	Unidentified	Drought and salinity	-	Enhance drought and salt tolerance	[53]
	*Talaromyces*	Unidentified	Sanity and oxidative stress	-	-	[54]
Pezizomycotina Incertae sedis	*Acrophialophora*	*Acrophialophora* *fusispora*	High temperature	-	-	[55]
Sordariomycetes	*Fusarium*	*Fusarium solani*	Drought	-	Promote plant growth under drought	[56]
	*Gibberella*	*Gibberella intricans*	-	-	-	-
	*Humicola*	*Humicola phialophoroides*	Salinity	-	Control of plant diseases/enhance growth in salt stress	[57,58,59]
	*Trichoderma*	unidentified	Drought	-	Enhance drought tolerance	[60,61]
	Others	*Hypoxylon anthochroum*, *Hypoxylon monticulosum*, *Nigrospora oryzae* and *Xylaria apiculata*	-	-	-	-
Ustilaginomycetes/ Agaricomycetes/ Cystobasidiomycetes	*Pseudozyma*	*Pseudozyma hubeiensis*	-	-	-	-
	*Coprinellus*	*Coprinellus aureogranulatus*	-	-	-	-
	*Gymnopilus*	*Gymnopilus dilepis*	-	-	-	-
	*Phellinus*	*Phellinus noxius*	-	-	-	-
	*Cystobasidium*	Unidentified	-	-	-	-

* Note:—stand for no data available.

## Data Availability

Raw sequence datasets are openly available in the National Center for Biotechnology Information (NCBI) under BioProject accession number PRJNA753229.

## Supplementary Materials

The following are available online at www.mdpi.com/article/10.3390/jof8100987/s1, Figure S1: Rarefaction curve of observed fungal OTUs across three study sites., Figure S2: Co-occurrence network of fungal OTUs detected in harsh environment of mining and rehabilitation sites, highlighting (a) taxonomic distribution at genus level and (b) functional distribution based on FungalTrait., Table S1: List of tree species found in the reference forest., Table S2: List of planted tree species in rehabilitation sites., Table S3: Method used for soil physicochemical measurement., Table S4: Goodness-of-fit (R^2^) and significant value (*p*-Value) of soil parameters fitted to NMDS ordinations of living fungal community, Table S5: Topology and node properties of co-occurrence network of fungal OTUs detected in degraded environment of mining and rehabilitation plots. Table S6: Living fungal taxa found in forest, mining, and rehabilitation plots.
