# Supplementary material for: Living Fungi in an Opencast Limestone Mine: Who Are They and What Can They Do?
Source: J Fungi (Basel). 2022 Sep 20;8(10):987. doi: 10.3390/jof8100987 (PMC9604801; doi:10.3390/jof8100987)
Supplement: Supplementary file 1 [file jof-08-00987-s001.zip › Supplemetary Figures.pdf]

Supplementary Materials

Living fungi in an opencast limestone mine: who are they and what they can do?

Chakriya Sansupa [1], Witoon Purahong [2,*], Ali Nawaz [3,4], Tesfaye Wubet [3,5], Nakarin Suwannarach [1,6], Panuwan Chantawannakul [1], Sutthathorn Chairuangsri [1], Terd Disayathanoowat [1,6,7,*]

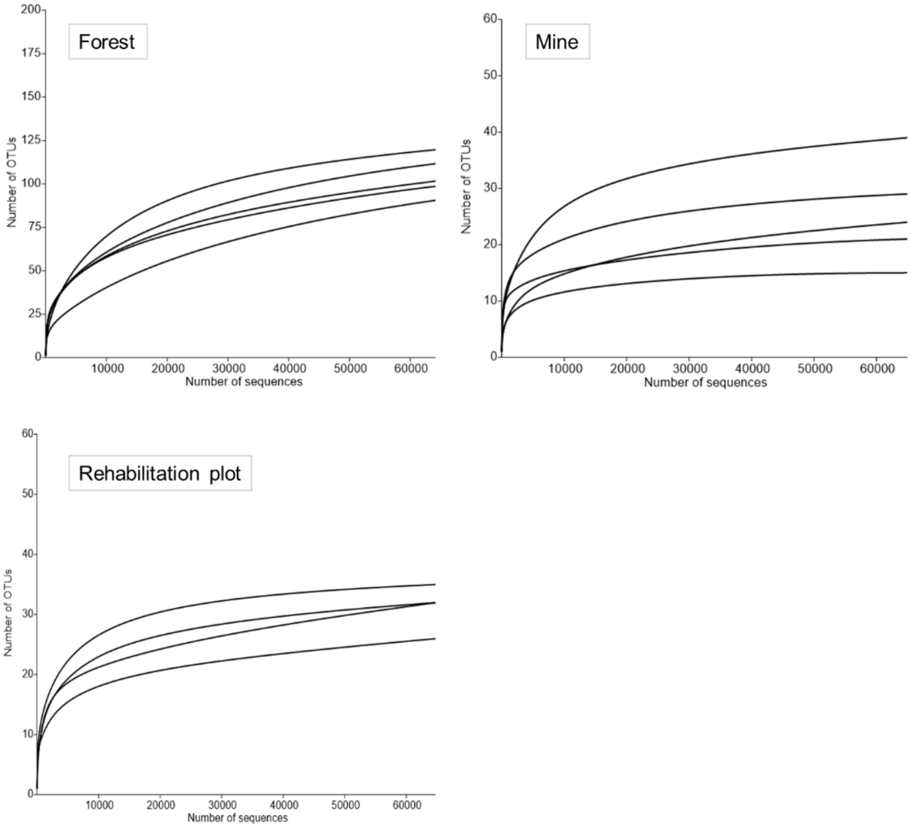

Figure S1. Rarefaction curve of observed fungal OTUs across three study sites.

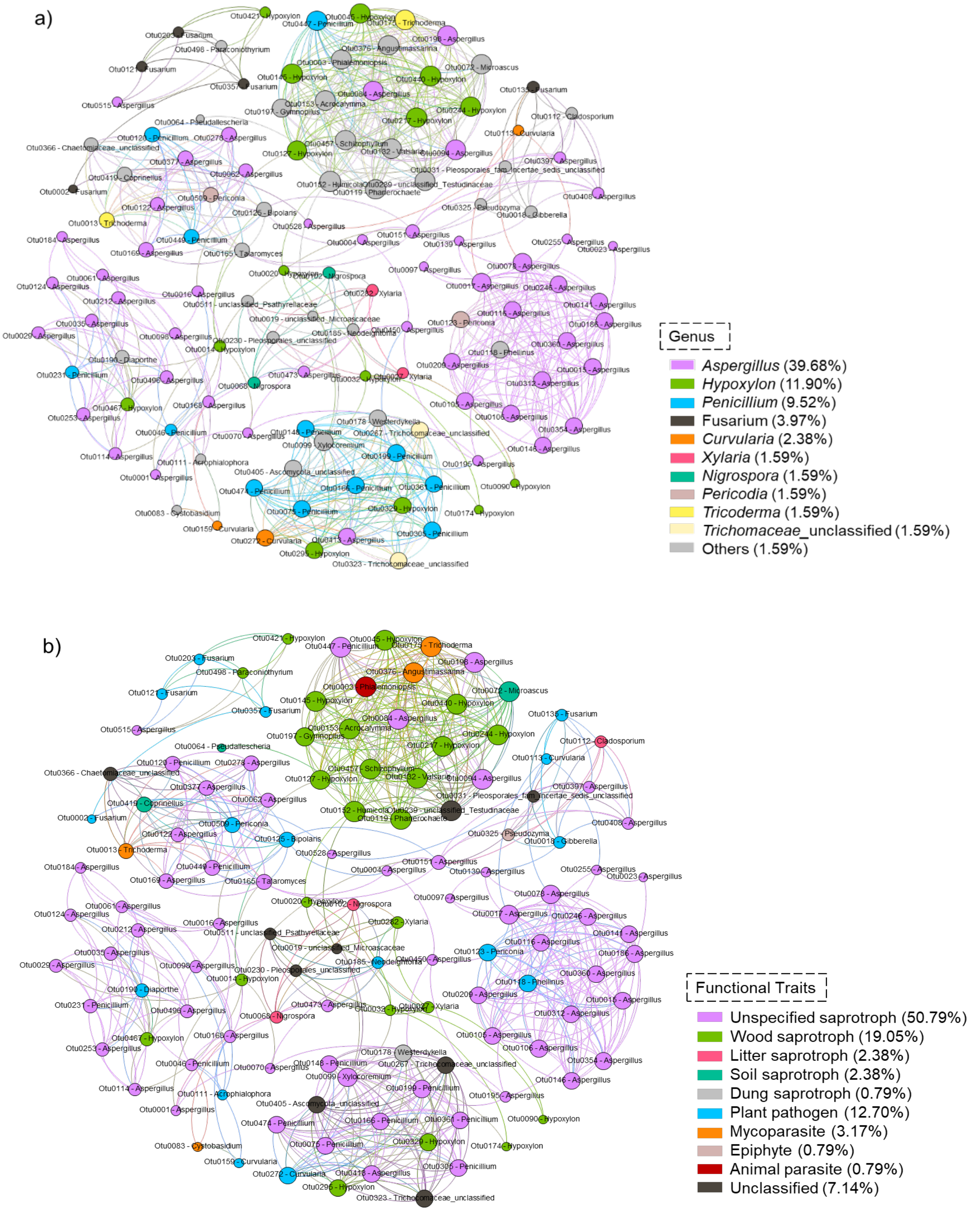

Figure S2. Co-occurrence network of fungal OTUs detected in harsh environment of mining and rehabilitation sites, highlighting (a) taxonomic distribution at genus level and (b) functional distribution based on FungalTrait.
